# Mediators of Host–Microbe Circadian Rhythms in Immunity and Metabolism

**DOI:** 10.3390/biology9120417

**Published:** 2020-11-25

**Authors:** Katya Frazier, Mary Frith, Dylan Harris, Vanessa A. Leone

**Affiliations:** 1 Department of Medicine, University of Chicago, Chicago, IL 60637, USA; katyafrazier@uchicago.edu (K.F.); mary.frith@uchospitals.edu (M.F.); dylanh@uchicago.edu (D.H.); 2 Medical Scientist Training Program, University of Chicago, Chicago, IL 60637, USA; 3 Department of Animal and Dairy Sciences, University of Wisconsin-Madison, Madison, WI 53706, USA

**Keywords:** circadian rhythms, metabolism, gut microbiota, immune function, microbial metabolites

## Abstract

**Simple Summary:**

Circadian rhythms serve as the body’s internal metronome, driving responses to environmental cues over a 24-h period. Essential to nearly all life forms, the core circadian clock gene network drives physiological outputs associated with metabolic and immune responses. Modern-day disruptions to host circadian rhythms, such as shift work and jet lag, result in aberrant metabolic responses and development of complex diseases, including obesity and Type 2 Diabetes. These complex diseases are also impacted by interactions between gut microbes and the host immune system, driving a chronic low-grade inflammatory response. Gut microbes exhibit circadian dynamics that are closely tied to host circadian networks and disrupting microbial rhythmicity contributes to metabolic diseases. The underlying mediators that drive communication between host metabolism, the immune system, gut microbes, and circadian networks remain unknown, particularly in humans. Here, we explore the current state of knowledge regarding the transkingdom control of circadian networks and discuss gaps and challenges to overcome to push the field forward from the preclinical to clinical setting.

**Abstract:**

Circadian rhythms are essential for nearly all life forms, mediated by a core molecular gene network that drives downstream molecular processes involved in immune function and metabolic regulation. These biological rhythms serve as the body’s metronome in response to the 24-h light:dark cycle and other timed stimuli. Disrupted circadian rhythms due to drastic lifestyle and environmental shifts appear to contribute to the pathogenesis of metabolic diseases, although the mechanisms remain elusive. Gut microbiota membership and function are also key mediators of metabolism and are highly sensitive to environmental perturbations. Recent evidence suggests rhythmicity of gut microbes is essential for host metabolic health. The key molecular mediators that transmit rhythmic signals between microbes and host metabolic networks remain unclear, but studies suggest the host immune system may serve as a conduit between these two systems, providing homeostatic signals to maintain overall metabolic health. Despite this knowledge, the precise mechanism and communication modalities that drive these rhythms remain unclear, especially in humans. Here, we review the current literature examining circadian dynamics of gut microbes, the immune system, and metabolism in the context of metabolic dysregulation and provide insights into gaps and challenges that remain.

## 1. Introduction

Circadian rhythms (CRs) provide essential internal signals that enable organisms to incorporate environmental light–dark signals into the temporal organization of physiology [1]. The circadian clock is a highly-conserved molecular feedback loop of transcription factors that direct the roughly 24-h cycle of clock-controlled genes and downstream processes. While several of the underlying mechanisms and responses to light–dark signals have been thoroughly examined, particularly how CRs direct host metabolic processes in various tissues, it is also clear that CRs are key for directing and maintaining rhythmicity in a myriad of immune cells throughout the body that aid in development of immune tolerance, antibody responses, and prevention of infection [2,3]. More recently, important connections between host CRs and the trillions of microbes that live in and on the body, termed the microbiome, have become evident and appear to be essential for orchestrating key host metabolic outcomes and homeostasis [4]. But how does this host–microbial orchestration take place and what factors aid in maintaining this rhythmic connection?

A recent body of work suggests that CRs associated with the immune system may serve as an important conduit for the relay of biological rhythms between host and microbe, driving both local host intestinal and peripheral tissue metabolic responses. Identification of key features that promote this essential cross-talk between host and microbe CRs, whether dietary, host, or microbially-derived, is crucial for the ability to translate these important findings to humans in order to promote immune-mediated metabolic health and wellness. In this review, we outline the current status of research revealing how host CRs are tightly integrated with rhythms in gut microbes, immune function, and metabolism, and we discuss unanswered questions that could be key to pushing the field forward.

## 2. Circadian Networks: Key Players that Drive the Metronome of Life

**Figure 1:**
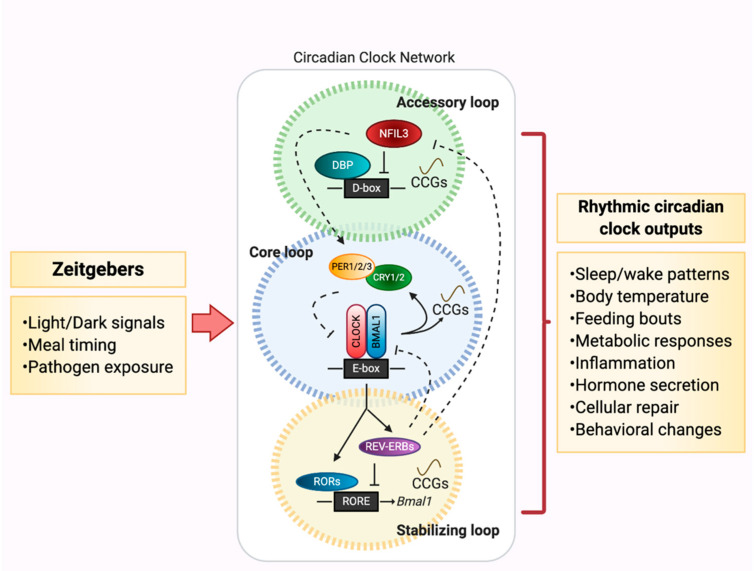
Circadian networks set the metronome of life. The highly conserved molecular circadian gene network is made up of a core negative feedback loop of transcription factors that respond to environmental Zeitgebers over a ~24 h period. The feedback loop consists of two primary activators (CLOCK and BMAL1), that dimerize and bind to E-Box promoter regions which induces transcription of clock-controlled genes (CCGs), including repressors Period 1-3 (PER) and Cryptochrome 1/2 (CRY). These proteins heterodimerize to prevent further transcription induction by CLOCK:BMAL1. Stabilizing loop components contribute to molecular clock regulation, including retinoic acid-related orphan nuclear receptors REV-ERBα and RORα, which induce vs. repress *Bmal1* transcription via RORE promoter binding. The accessory loop is made up of the activator albumin-D-box binding protein (DBP) and repressor nuclear factor interleukin 3 (*Nfil3*), which influence *Per* gene expression via D-box promoter binding. These loops drive and maintain rhythmic expression of the underlying circadian clock network and each contributes to overlapping and mutually exclusive sets of CCGs that drive rhythmic physiological responses.

The molecular machinery that aids in maintaining CRs throughout the body consists of a gene network that is highly conserved across invertebrate and vertebrate species. At the molecular core of the circadian clock is a negative feedback loop of transcription factors expressed in nearly every tissue [5], consisting of two primary activators, Circadian Locomotor Output Cycles Kaput (CLOCK) and Brain and Muscle ARNT-Like 1 (BMAL1), which dimerize to form the transcription factor complex CLOCK:BMAL1. This complex binds to E-Box promoter regions to induce gene transcription of clock-controlled genes (CCGs). Among these CCGs are feedback loop repressors Period 1-3 (PER) and Cryptochrome 1/2 (CRY), which heterodimerize and cause dissociation of CLOCK:BMAL1 from DNA-binding sites, preventing further induction of transcription. PER and CRY are then ubiquitinated and degraded, thus resulting in re-initiation of the ~24-h molecular circadian clock negative feedback cycle. Secondary components contribute to the molecular clock regulation, including retinoic acid-related orphan nuclear receptors REV-ERBα and RORα, which respectively induce and repress *Bmal1* transcription via promoter binding. Finally, tertiary, but equally important factors are the activator albumin-D-box binding protein (DBP) and repressor nuclear factor interleukin 3 (*Nfil3*, otherwise known as E4BP4), which influence *Per* gene expression [6]. Together, these three loops drive and maintain rhythmic expression of the underlying circadian clock network (Figure 1).

While nearly all tissues express circadian clock transcriptional factors, the central pacemaker is located within the suprachiasmatic nucleus (SCN) of the hypothalamus, serving as a key sensor of light:dark (LD) signals and whose rhythms entrain the ~24-h cycle in nearly all peripheral cell clocks through a variety of rhythmic endocrine and humoral cues, many of which are still under investigation. Peripheral clocks are located throughout the body, ranging from the largest metabolic organ, the liver, to adipocytes, to intestinal epithelial cells, to leukocytes that traffic throughout the body [7]. Together, coordination of both central and peripheral clocks drives organismal expression of molecular, behavioral, and thermoregulatory CRs essential for life, aiding in refined responses to environmental perturbations.

## 3. Circadian Rhythms Gone Astray: A Defunct Metronome

Alterations or breaking of the host clock, either by aberrant environmental signals or genetic manipulation of circadian clock gene expression, results in emergence of a wide variety of atypical physiological outcomes, including metabolic disturbances. Metabolic syndrome, which encompasses a cluster of conditions including increased blood pressure, Type 2 Diabetes (T2D), and obesity, is rapidly becoming a world-wide health epidemic. Although many factors contribute to metabolic disorders, such as an overt increase in caloric intake and decreased energy expenditure, internal circadian disorganization significantly impacts metabolism. For example, insufficient or irregular sleep due to shift work or jet-lag alters entrainment to the light–dark cycle, leading to circadian dysregulation mediated by disturbances in core circadian clock genes; these lifestyle factors are consistently associated with increased obesity risk [8]. In addition to environmental circadian disruption, countless animal models demonstrate that loss of a core circadian clock gene results in a wide array of negative health outcomes ranging from aberrant body weight and adiposity to insulin resistance [9].

Although peripheral circadian clocks are certainly directed by rhythmic signals from the SCN, they also govern unique aspects of physiology that largely depend on the primary tissue function. For example, mice globally deficient in *Bmal1* exhibit vast physiological issues, including arrhythmic activity [10], low bone mass [11], rapid aging [12], as well as abnormal glucose [13] and lipid [14] homeostasis coupled with insulin resistance. However, when *Bmal1* is deficient only in the liver [15], many of these abnormalities are absent and mice instead have an opposite, unique glucose tolerance phenotype. Similar patterns have been observed with other murine tissue-specific deletions; loss of *Bmal1* from pancreas results in severe insulin insensitivity [16], while adipocyte-deficiency results in obesity and altered food intake patterns [17]. Circadian clock expression within immune cells also induces changes in metabolic outcomes. In addition to impacting infection clearance ability, loss of *Bmal1* only in monocytes also induces a significant increase in weight gain when given high-fat diet (HFD) compared to wild-type (WT) controls [18]. Tissue-specific *Bmal1* rescue provides further evidence of peripheral clock autonomy. *Bmal1* rescue only in the brain of globally-deficient mice successfully restores wheel-running activity, but overall reduced activity levels persist, while *Bmal1* rescue in muscle elicits opposing effects [19]. The complexities of tissue-specific host circadian clock gene autonomy vs. interactions with one another in conjunction with physiological outcomes is not fully understood.

While circadian disturbances often result in altered metabolic outcomes, inflammation and the immune system at large plays a major role in this relationship and the general development of metabolic disorders [20]. For example, obesity is often characterized by chronic low-grade inflammation resulting from continuous activation of the immune system, a hallmark of which is low-grade endotoxemia, or exposure to constant, low levels of lipopolysaccharide (LPS) derived from gram-negative intestinal bacteria [21]. Inflammatory cytokines such as tumor necrosis factor-alpha (TNFα), IL-1β, and IL-6 can cause insulin resistance when overexpressed in adipocytes, which typically occurs in insulin resistant, non-metabolically healthy obese as compared to metabolically healthy obese individuals who retain insulin sensitivity [22,23,24]. The mechanisms differentiating metabolically healthy vs. un-healthy individuals are unclear; however, a lack of inflammation in metabolically healthy obese individuals may prevent insulin resistance, which has been correlated with individual chronotype. Indeed, metabolically unhealthy, obese individuals experience increased social jetlag (defined as differences between the social clock imposed by societal norms, such as work or school schedules, and the internal biological clock) which associates with increased co-morbidities including high blood pressure, high waist circumference, low HDL, high circulating triglycerides, and high glycated hemoglobin coupled with high C-reactive protein, which would indicate increased inflammation [25]. Aside from social jet lag, additional factors, both internal and environmental, that drive healthy vs. unhealthy obesity remain elusive, however, shifts in gut microbiota may play a role as discussed below.

## 4. Gut Microbes: A Novel Instrument in the Orchestration of Circadian Rhythms

The mammalian GI tract is colonized by a population of ~10^14^ bacteria that becomes exponentially more complex and diverse along the horizontal axis from mouth to anus, where each intestinal region harbors a unique microbial community [26]. The gut microbiome’s role in promoting host health is extensive; however, gut microbial dysbiosis induced by intrinsic and extrinsic factors can push disease development in genetically-prone individuals. HFD selects for unique microbial community membership, particularly taxa belonging to the dominant phyla Firmicutes and Bacteroidetes, with corresponding alterations in the microbiome’s functional capacity [27]. These shifts have been directly linked to obesity and inflammation. When microbes from HFD-fed obese mice are transferred into a germ-free (GF) host completely devoid of all microbes, the conventionalized recipients develop obesity despite continued intake of a low fat, high fiber diet [27], suggesting specific microbial communities can drive metabolic disruption. One modality of gut microbiota-host cross-talk is through a conserved group of receptors classically involved in innate immunity, termed Toll-Like Receptors (TLRs) [28], which recognize common microbe-associated molecular patterns (MAMPs), such as LPS, peptidoglycan, and flagellin. TLR expression exhibits circadian dynamics that are dependent, in part, on gut microbes and their activation can result in downstream induction of pro-inflammatory cytokines, including TNF-α, IL-6, and IL-1β [29], driving obesity-associated inflammation. The role of gut microbes in facilitating normal CRs as well as their involvement in CR disruption mediated by aberrant host immune signaling resulting in negative metabolic consequences is just beginning to be explored. Here, we discuss what is currently known about how CRs and gut microbiota interact with and influence one another in the context of immune function and metabolism.

### Gut Microbiota, Circadian Rhythmicity, and the Host: A Conserved Orchestration

Host–microbe crosstalk in the context of biological rhythms is well conserved across vertebrates and invertebrates, as exemplified by the symbiotic interactions between the Hawaiian sepiolid squid *Euprymna scolopes* and its bacterial symbiont *Vibrio fischeri*. *V. fischeri* colonizes the crypts of the squid’s light organ shortly after birth, outcompeting more abundant bacteria to become the exclusive bacterial symbiont, whose bioluminescent properties help to camouflage the squid from predators. Rhythmic colonization and expulsion of *V. fischeri* by the squid contributes to development and maintenance of the squid’s light organ, the expression of cryptochrome genes [30], and transcriptional oscillation of certain metabolic gene programs, which is abolished in the absence of *V. fischeri* [31].

The host innate immune responses to bacterial signals from *V. fischeri* seem to be key to maintain these host–microbe interactions. Ciliated epithelial cells and macrophage-like haemocytes recognize MAMPs, including LPS and peptidoglycan from both *V. fischeri* and other bacteria [32,33]. However, *V. fischeri*, unlike other bacteria, is able to enter the light organ crypts to evoke these responses through several co-evolved strategies that maintain squid-vibrio symbiosis. For example, chemotaxis of *V. fischeri* toward N-acetylneuraminic acid in host mucus attracts these bacteria to the vicinity of *E. scolopes* [34]. Once colonized, *V. fischeri* exhibits diel switching between anaerobic respiration and anaerobic fermentation, rhythmically altering the pH within the light organ [35,36]. This symbiosis between *E. scolopes* and *V. fischeri* serves as a simplified model system of host–microbe interactions relative to more complex communities, such as those within the mammalian gastrointestinal (GI) tract [33].

The mammalian intestine represents an intricate network of circadian host–microbe interactions, as it harbors the largest numbers of microbes. As in the squid-vibrio example, the mammalian host interacts with resident gut microbes largely by way of the innate immune system, albeit in a more complex manner. This includes pathogen recognition receptors (PRRs) such as TLRs, Nod-like receptors, Aryl hydrocarbon receptors, and nuclear receptors such as peroxisome proliferator-activated receptors (PPARs), each exhibiting their own diurnal expression patterns [37]. For instance, LPS and flagellin are recognized by TLRs, many of which act through the signal transduction adapter myeloid differentiation primary response 88 (MyD88) expressed in a myriad of host tissues, ranging from the intestinal epithelial cell compartment to immune cells involved in antigen presentation, such as dendritic cells. Several studies have shown that under normal conditions, ~15% of bacterial taxa exhibit diurnal oscillations in microbial community membership over a 24-h period in rodents, as determined via 16S rRNA gene sequencing as well as functional outputs that result in increased host exposure to microbially-derived components [38,39,40]. Additionally, similar to that observed in the squid, gut microbes oscillate in their proximity to the intestinal mucosa in mice, which appears to be primarily driven by timing of host feeding [40]. Perturbing diurnal rhythmicity of gut microbes dysregulates host CRs, and reciprocally, the gut microbiota is sensitive to circadian disruptions such as changes in diet, time of eating, and sleep-wake patterns as discussed below.

## 5. Microbe vs. Host-Mediated Control of Circadian Orchestrations: Who Conducts Who?

### 5.1. Microbe-Mediated Signals Conduct Host Circadian Rhythms

Both the presence and composition of bacteria are important for host–microbe diurnal cross-talk, as well as for the promotion of normal host circadian network functionality. Experimentally, examination of the functional role of gut microbes in vertebrate mammalian systems generally involves the use of GF mice (or those colonized with select microbial communities), dietary alterations, environmental disturbances, or antibiotics. In general, GF mice are resistant to HFD-induced obesity, produce lower levels of inflammatory cytokines including TNFα, IFNγ, IL-1B, and IL-17 [41], and have blunted transcriptional oscillations of the core clock gene *Bmal1* [38] relative to conventionally-raised specific pathogen-free (SPF) mice. This underscores the importance of gut microbes in driving host molecular circadian gene expression. Conventionalization of GF mice increases insulin resistance, lipid absorption, fat mass, and de novo lipogenesis in the liver [42], and also restores diurnal transcription of genes involved in these processes [43]. HFD alters oscillation patterns of hepatic *Bmal1* gene expression in both GF and SPF mice, reducing the abundance of oscillating bacterial taxa while increasing inflammation in mesenteric adipose tissue in the latter [38,44]. Furthermore, colonizing GF mice with dysbiotic microbes from jet-lagged murine and human hosts increased insulin resistance and weight gain relative to mice colonized with microbes from non-jet-lagged counterparts [40]. Microbial depletion via antibiotics (ABX) in SPF mice abolishes oscillatory transcriptional patterns of many host genes, such as those involved in innate immune signaling [37], as well as lipid oxidation and catabolism, while provoking de novo oscillation of genes involved in fatty acid and amino acid metabolism, suggesting metabolic compensation by the host in the absence of microbial cues [40]. Together, these studies demonstrate ways in which gut microbes have evolved to interact with and affect host immune function in the context of CRs and metabolism.

### 5.2. Disrupting the Tempo of Circadian Rhythmicity: Host Circadian Network Perturbations and Gut Microbial Oscillations

Numerous metabolic and immune processes exhibit CRs under normal conditions and can be severely impacted by host central or peripheral clock manipulation. A total of 43% of protein-coding genes display oscillating transcription patterns in at least one organ or tissue, with significant variations depending on tissue type [45]. In particular, oscillating CCGs within the liver significantly impact metabolic and insulin signaling pathways [46]. Many metabolic processes and programming are induced by meal timing, and as a result, exhibit corresponding rhythms centered around food intake. Similarly, infection rates and severity vary over a 24-h period, in part due to the rhythmic oscillation of several innate and adaptive immune components, including PRRs, cytokine production, and immune cell proliferation and activity [47]. For example, sepsis severity depends upon and correlates with diurnal expression and functional patterns of TLR9 [48]. These oscillations are particularly sensitive to CR disruption within the host, either by changes to the environment or through genetic manipulation, as discussed herein. The outcomes of CR disruption are also summarized in Table 1, with additional references.

### 5.3. Environmental Perturbations Disrupt Orchestration of Circadian Rhythms

#### 5.3.1. Jet Lag and Shiftwork

Epidemiological studies suggest constant circadian misalignment, such as in individuals performing shift work or experiencing regular jet lag, is significantly associated with metabolic disease incidence [85]. Jet lag can be experimentally induced in animal models by phase-shifting the LD schedule either forward or backward. Mice exposed to shifts in light exposure exhibit a significant increase in overall body weight and adipose tissue with corresponding insulin insensitivity compared to non-jet-lagged animals, as well as abnormal sleep and feeding patterns regardless of dietary fat content [40,51]. Frequent time shifts have also been shown to induce major alterations in overall gut microbiota community membership and a loss of oscillation in specific taxa [40,51], particularly when exposed to HFD [49]. Additionally, depletion of gut microbes via ABX diminishes the host’s metabolic responses to experimentally-induced jet lag [40], perhaps mediated by loss of functional microbial outputs. Under conditions of phase advanced jet lag, treatment with short chain fatty acids (SCFAs, key microbial products of fermentation that can directly signal to the host) significantly accelerated entrainment of host peripheral clocks [86]. Microbial genes involved in SCFA production are also downregulated in response to constant light exposure [52].

Shift workers are also more susceptible to bacterial and viral infections [87,88], which has been explored in mice. In addition to confirming previous metabolic and microbial outcomes following altered light exposure, jet lag induced elevated levels of inflammatory macrophages and decreased T regulatory cells in white adipose tissue [51]. Further, jet lag in mice results in elevated levels of intestinal Th17 cell populations [54], as well as endotoxemia coupled with significantly higher mortality rates following LPS exposure [55]. Jet lag conditions have also been shown to increase overall gut permeability [49,50], which would result in more direct exposure of the host to gut microbial products eliciting an elevated immune response. Together, altered light exposure and sleep patterns via jet lag or shift work greatly impact host metabolism as well as immune responses in the intestine and peripherally and may be influenced by gut microbes.

#### 5.3.2. Time Restricted Feeding

Time-restricted feeding (TRF) as an experimental manipulation consolidates feeding bouts, and accordingly, alters activity patterns. As a result, TRF impacts immune responses to pathogens, where mice infected with the parasitic helminth *Trichuris muris* could expel the worms significantly faster if infected during the rest (light) phase as compared to infection during the active (dark) phase, for which *Bmal1* expression on dendritic cells was essential [60]. However, TRF to the rest phase resulted in a loss of more rapid infection clearance. Similarly, rest phase TRF significantly reduced peripheral cytokine production in serum and bacterial killing capacity in response to active phase LPS challenge [58], while active phase TRF increased production and expression of these innate immune factors.

TRF alters or resets internal circadian dynamics, particularly in peripheral metabolic organs such as the liver, even in absence of a functional central clock [89]. Mice fed HFD ad libitum exhibit sporadic feeding patterns throughout the LD cycle, resulting in amplitude and periodicity dampening of host circadian elements in the liver, including decreases in *Per2*, *Bmal1*, *Rev-erbα*, and *Cry1* due in part to perturbed AMP-activated protein kinase (AMPK) levels.

TRF only to the active dark cycle can restore diurnal oscillations in the liver, as well as improve host metabolism and protect against HFD-induced obesity [56]. While active phase TRF reduces negative metabolic outcomes of HFD, adipose tissue inflammation and macrophage infiltration is not prevented [57], implying these immune and metabolic outcomes are regulated by different mechanisms. Conversely, TRF to the rest phase in mice disrupts natural sleep and behavioral patterns, resulting in major disruptions of host gene expression, particularly in liver, independent of the central clock [59]. Gut microbiota are also particularly sensitive to meal timing. TRF of HFD to the active phase in mice partially restored gut microbial composition associated with HFD, as well as a portion of diurnal oscillations in microbial relative abundance [39]. Restoration of gut microbiota rhythms, despite HFD exposure, may partially explain how TRF restores host rhythms and prevents diet-induced obesity. Together, peripheral circadian disorganization due to altered feeding patterns coupled with irregular metabolic cues impacts the host’s ability to mount an appropriate immune response which could be mediated, in part, by circadian dynamics of gut microbes.

#### 5.3.3. Molecular/Genetic CR Disruption: Circadian Knockout Models

Genetic ablation of core circadian clock genes, either globally or in specific tissues, is used to examine how loss of a functional internal clock impacts physiology. Similar to experimental jet lag, global *Clock* mutant mice also exhibit increased gut permeability [50], which may result in increased exposure of the host to gut microbiota, altering immune-mediated responses to microbial invaders throughout the body. For example, global *Bmal1* deficient mice are more susceptible to infection [63] and selective *Bmal1* deletion from ILCs results in reduced ILC cellularity in the intestine [65]. The diurnal dependency of helminth expulsion by the host is also dependent upon *Bmal1* within dendritic cells [60]. While certain immune cells and related factors normally exhibit diurnal patterns over 24-h, *Bmal1* deficiency specifically from myeloid cells [18] or neutrophils [66] abolishes their rhythmic trafficking and impairing the host’s ability to clear infection. Rhythmic trafficking of lymphocytes is also dependent on *Bmal1* [64]. These phenomena persist in absence of additional circadian genes; *Per2* or *Per1* deficient mice exhibit loss of daily splenic and circulating IFN-γ rhythms [79], with corresponding loss of rhythmic cytolytic natural killer cell factors [80] in the latter. *Clock* expression levels directly correlate with dermatitis outcome in mice driven in part by CLOCK promotor binding and induction of IL-23 receptor expression in gamma/delta T cells, which contributes to diurnal *IL-23* receptor expression patterns under basal conditions [69].

The striking effects of genetic core clock deficiency are not only unique to the immune system, but also apparent in metabolism and gut microbiota interactions. Extensive published work shows the metabolic effects of global genetic circadian gene deficiency in clock genes such as *Clock*, *Bmal1*, *Per1/2*, and others. For example, global *Clock* mutant mice gain significant amounts of body weight and display increased food intake on either regular chow or HFD [68]. Global deficiency of *Bmal1*, results in a variety of metabolic abnormalities, including insulin insensitivity and aberrant glucose and lipid homeostasis [5,14,62,90,91,92]. Global mutants of other clock genes exhibit unique metabolic outcomes; *Cry1*-deficient mice are uniquely resistant to HFD-induced obesity, while *Cry2*-deficient mice remain susceptible [93]. Interestingly, *Cry1/2*-double knockout mice gain significantly more weight when challenged with HFD than WT counterparts, coupled with hyperinsulinemia and increased lipid uptake [71]. Global loss of *Per2/3* [75,76,77,78] or and *Rev-erb α/β* [82] also induces unique metabolic characteristics. Together, global core clock gene deletion not only demonstrates their essential role in normal metabolic health and homeostasis, but also the unique effects each component elicits in maintaining normal physiology.

Genetic deletion of core circadian clock components also induces significant changes in gut microbiota composition. For instance, global deletion of both *Per1/2* in mice changes gut microbiota community membership and a loss of oscillating taxa, particularly those belonging to the phyla Proteobacteria and Bacteroidetes [40]. Global *Clock* [67] or *Bmal1* [61] mutant mice also exhibit significant changes in overall fecal microbiota taxonomic composition as well as reduced taxonomic diversity. Interestingly, oscillating microbes display a sex-dependency, where female mice exhibit more significant diurnal oscillations in fecal bacteria relative to males, and each sex induces a unique set of microbial oscillators [61]. It is clear that host expression of specific clock genes affects fecal microbiota oscillations and more work is required to delineate the specific effects that each core clock component, whether central or peripheral, elicits on gut microbiota, along the length of the GI tract. These shifts may be mediated by a combination of molecular mechanisms and changes in behavior and feeding patterns.

Transcription factors involved in the third, accessory loop of the circadian gene network are equally important to maintaining broader host circadian dynamics in metabolism and immunity. *Nfil3* diurnal gene expression amplitude in intestinal epithelial cells (IECs) is mediated by the presence of gut microbes, where GF mice exhibit nearly arrhythmic expression that correlates with their protection against HFD-induced obesity. IEC-specific *Nfil3* deletion in SPF mice revealed a nearly identical phenotype to GF counterparts [83]. These effects appeared to be mediated by a MyD88-dependent signaling relay initiated by gut microbes through innate immune cells and group 3 Innate lymphoid cells (ILCs) that ultimately influence the core circadian clock component *Rev-erbα* and downstream induction or suppression of *Nfil3*, driving host metabolism [83]. Here, gut microbes engage both circadian and immune components to direct host metabolism.

Together, these studies demonstrate that both the core circadian clock and gut microbes are essential for normal metabolic as well as innate and adaptive immune responses. The intersections between these systems and how they direct metabolism and immunity are multifactorial and complex, relying on a variety of signaling factors to direct physiological outcomes, outlined below.

## 6. Transmitting the Metronome’s Rhythm: Molecular Mediators of Host–Microbe Interactions

**Figure 2:**
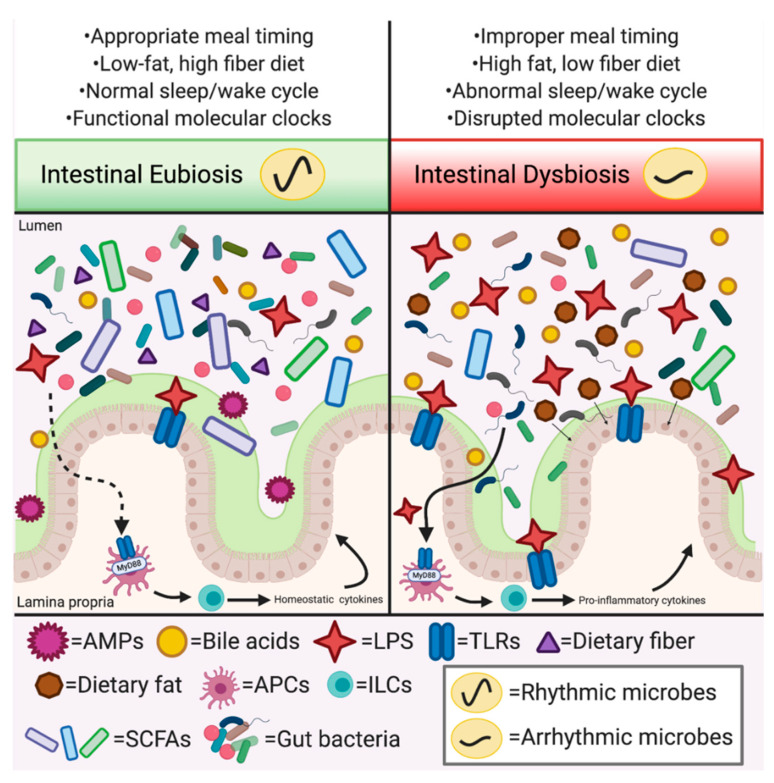
Mediators of circadian orchestration between environment, gut microbes, and the immune system that contribute to metabolic dysregulation. Environmental stimuli and host behavioral outputs contribute to intestinal eubiosis vs. dysbiosis. For instance, low-fat, high fiber diets contribute to eubiosis characterized by increased microbial diversity exhibiting diurnal oscillations in membership and function (e.g., SCFA production). Diurnal stimulation and release of AMPs mediated by TLR stimulation on both epithelial and immune cells, such as antigen presenting cells (APCs) and innate lymphoid cells (ILCs), coupled with normal cytokine production aid in maintaining the mucosal layer and epithelial barrier, preventing overexposure to LPS and other microbially-derived products. High fat diet (HFD) or other circadian disruptions lead to intestinal dysbiosis, characterized by decreased microbial diversity and a loss of microbial oscillators with decreased functional outputs, such as SCFAs. A HFD shifts bile acid profiles and elevates LPS-producing bacteria resulting in a thinner mucosal layer, epithelial barrier dysfunction and increased stimulation of APCs and ILCs leading to heightened production of pro-inflammatory cytokines, contributing to metabolic dysregulation and disease.

Beyond timing of feeding, what endogenous compounds facilitate and regulate circadian host–microbe metabolic and immune interactions? While further exploration is needed to fully answer this question, several studies implicate SCFAs, LPS, bile acids, AMPs, and melatonin as critical effector molecules in these complex and multifaceted interactions, as outlined below and summarized in Figure 2.

### 6.1. SCFAs

SCFAs, which include butyrate, propionate, and acetate, are bacterial metabolites primarily produced via fermentation of dietary carbohydrates that are indigestible by host enzymes. SCFAs have multifaceted activities as endocrine and immune effector molecules, with roles in nutrient sensing, lipid absorption, gluconeogenesis, and insulin secretion [94]. SCFAs oscillate in vivo and regulate host core clock gene expression [38]. Under regular, low-fat chow feeding conditions in mice, many taxa exhibiting oscillations belonged to those involved in fermentation and SCFA production; under HFD, fermentative bacterial oscillations and their SCFA levels were lost [38]. Providing causal evidence for a role of butyrate in modulating host CRs, intraperitoneal injection of butyrate into GF mice shifted the *Per2:Bmal1* gene expression ratio in the liver relative to vehicle-injected controls. In vitro studies revealed similar results, where butyrate treatment of hepatic organoids increased amplitude of *Bmal1* and *Per2* oscillations relative to vehicle control. These studies revealed products derived from diet-induced gut microbes can directly impact CRs in peripheral organs.

SCFAs have a multitude of activities in vivo but the most relevant to metabolism and immunity may be their stimulation of free fatty acid receptors (FFARs) on enteroendocrine cells (EECs) and several immune cell types, their role as an energy source for IECs, as well as butyrate’s impact on gene expression via histone deacetylase (HDAC) inhibition. Upon stimulation of FFARs by SCFAs, EECs respond by releasing gut hormones including GLP-1, PYY, or 5-HT. However, the specific responses of these cells depend on the intestinal context, as hormone release is regulated by the core circadian clock, the position of the cell along the crypt-villus axis, and the host species (some studies indicate that EECs from humans versus mice respond differently to SCFA stimulation) [95,96,97,98,99,100]. Additionally, SCFAs exhibit unique effects on EECs; butyrate can stimulate serotonin (5-HT) release from Enterochromaffin cells (ECs) and accelerate intestinal motility [101], while acetate reduces serotonin receptor 5-HT_3_ expression on EC cells to modulate colonic fluid secretion [102], and a mixture of SCFAs stimulated release of GLP-1 and PYY and slowed intestinal motility [100]. Butyrate serves as the preferred energy source for IECs, and as such, mice with depleted butyrate-producing organisms (e.g., GF mice), demonstrate elevated autophagy of IECs and a weakened epithelial barrier [103]. Butyrate also has distinct anti-inflammatory actions on a number of immune cells via HDAC inhibition (reviewed elsewhere [103]).

High levels of SCFAs may reduce food intake and body weight by suppressing appetite [104,105] and reducing inflammation [94,106]. However, SCFAs also provide calories to the host upon absorption as lipids, promote insulin release, and are elevated in many obese individuals who often have concomitant low grade inflammation [94,107,108]. Therefore, the beneficial effects of SCFAs may be dose-dependent and/or specific to certain SCFAs and cell types. Other factors in obesity might also mask the beneficial effects of SCFAs for weight reduction and anti-inflammation. Although the mechanisms of how SCFAs impact inflammation and adipogenesis is unclear, their actions and influence on metabolic health underscore their overall importance to host physiology and represent one way of circadian host–microbe interaction.

### 6.2. Bile Acids

Bile acids are primarily synthesized in the liver from cholesterol and released into the intestinal lumen, where they aid in digestion of dietary fats. Normally, specific groups of microbes deconjugate bile acids (i.e., remove conjugated taurine or glycine residues), which leads to either resorption into hepatic circulation or excretion in stool. Bile acid synthesis follows a diurnal pattern [109] and is regulated by feeding patterns and the core circadian clock [110]. A negative feedback loop also exists in which bile acids inhibit their own synthesis, keeping the overall levels in check.

Evidence from multiple studies suggests bile acids impact CRs, metabolism, and immune function, and that the actions of bile acids are influenced by the gut microbiota [111,112,113]. In particular, bile acids affect glucose and lipid metabolism through bile acid signaling via FXR, reviewed by Dawson and Karpen [113]. GF mice, and to some extent ABX-treated mice, have a bile acid pool that contains higher levels of tauro-β-muricholic acid which inhibits FXR signaling. Since FXR signaling increases the expression of genes such as FGF15, which suppress the expression of *Cyp* genes such as *Cyp7A1* (the primary enzyme needed for bile acid synthesis from cholesterol), this could result in alterations to bile acid synthesis and CRs. Bile acids also can act as AMPs or stimulate release of other AMPs [113], suggesting a role in innate immune function. In this way, bile acids are important mediators in the complex interactions of gut microbes, host metabolism, host immune function, and host CRs.

Disrupting this complex system in different ways may modulate other components of the system. For instance, TRF of HFD in mice protected against DIO and resulted in significantly higher levels of primary bile acids in their stool, relative to HFD ad libitum-fed counterparts. TRF of HFD-fed mice also elicited lower abundance of *Lactobacillus* relative to either ad libitum HFD or regular chow-fed mice, which includes several species that express bile acid hydrolase genes [39]. In another study, bile acid synthesis was higher in GF mice fed palm oil-based HFD relative to GF mice fed lard-based HFD, without an observed difference in hepatic cholesterol and triglyceride levels between mice fed the two fat sources [114]. Furthermore, the normal diurnal rhythmicity of the respiratory exchange ratio was lost in mice fed palm oil-based HFD and only reduced in amplitude with the lard-based HFD. These findings suggest that changes in dietary patterns affect microbial composition, oscillation, and bile acid metabolism in the host, which may lead to metabolic changes partially mediated by disturbed bile acid signaling.

### 6.3. LPS

Microbial antigens such as LPS, a component of the cell wall of gram-negative bacteria, elicit immune-mediated effects on host metabolism. LPS recognition by TLRs, such as TLR4, can result in downstream activation of nuclear factor-kB (NF-κB) which leads to induction of a number of pro-inflammatory cytokines [29]. Previous work has revealed that time of exogeneous LPS exposure impacts immune-mediated outcomes both in mice and humans, where treatment during transition from periods of rest to activity drives stronger NF-κB activation and increased cytokine production [115]. Metatranscriptomic analysis of stool samples from circadian disrupted mice induced by constant light (LL) exposure revealed shifts in overall gut microbiota 16S rRNA gene alpha- and beta-diversity indices. This was coupled with altered functional characteristics, including increased expression of genes associated with LPS translocation (LPS export system permease protein [*LptF*]) and synthesis (3-Deoxy-manno-octulosonate cytidylyltransferase [*kdsB*]) that corresponded to increased gut permeability in ex vivo intestinal tissue relative to mice maintained in standard LD conditions [52]. Further, LPS binding protein (LBP), a surrogate marker for circulating LPS, was dramatically increased in obese children with obstructive sleep apnea (OSA) relative to obese children without OSA and nonobese control subjects, suggesting circadian disruption is associated with low-grade endotoxemia that may drive metabolic dysfunction [116]. Together, these studies suggest host circadian disruption can alter microbiome-mediated levels and exposure to LPS, contributing to low-grade inflammation and obesity.

The presence of LPS within the microbial community is important, since lack of a microbiome leads to loss of TLR diurnal expression patterns and subsequent alterations in local glucose and lipid homeostasis within the intestine [37]. Wang et al. (2017) elegantly demonstrated that LPS can exert an effect on metabolism through the immune and circadian systems, showing that LPS-induced TLR-mediated signaling through MyD88 on dendritic cells ultimately promotes *Nfil3* transcription and downstream expression of genes associated with lipid uptake, depending on the microbial context [83]. *Nfil3* oscillates diurnally within distal small intestinal IECs and requires presence of motile, gram-negative (i.e., LPS-containing) bacteria to fine-tune diurnal amplitude of IEC expression patterns, which ultimately impacts nutrient uptake in the gut. Reinforcing this notion, LPS treatment alone increased *Nfil3* expression in cultured IECs [83]. While this host–microbe crosstalk results in changes in *Nfil3* and lipid metabolism in the gut, the communication occurs further upstream and requires many cell types involved in mucosal innate and adaptive immunity. This is just one example where a microbial antigen, LPS, activated an immune response that affects host metabolism via CRs; others may have yet to be described. For instance, mono-colonization of GF mice with *Bacteroides thetaiotaomicron* upregulated Histone Deacetylase 3 (HDAC3) in a MyD88-dependent manner, where HDAC3 was required for diurnal nutrient uptake and lipid absorption in IECs [43], suggesting specific groups of bacteria and their specific mediators have distinct roles in circadian host–microbe interactions.

### 6.4. Antimicrobial Peptides (AMPs)

Host-derived AMPs serve as a primary modality to exclude pathogens from the microbial community and regulate commensal organisms. Changes in microbial composition can lead to shifts in AMP production, which often correspond with intestinal inflammation. Similarly, metabolic disruption can also shift intestinal AMPs [117]. Mice fed an HFD exhibited a shift in gut microbiota community membership that correlated with significant decreases in AMPs, such as *Reg3γ*, *lysozyme*, and *angiogenin 4*, while inflammatory cytokines, including TNF-α, IFNγ, IL-1β, and IL-6 were significantly upregulated in the small intestinal epithelium [118]. The AMP REG3γ inhibits epithelial colonization by gram-positive bacteria as part of an innate immune response via the lectin-mediated complement pathway [119]. REG3γ exhibits diurnal expression patterns that are regulated by the microbiome, where GF and ABX-treated mice exhibit an overall loss of diurnal *Reg3γ* expression [37,120]. One study revealed REG3γ is a component of the CCG network, mediated by *Rev-Erbα* and TLR4/STAT3 signaling through NF-κB [37], while additional work suggested specific microbial community members induced by a low-fat high fiber diet, specifically *Lactobacillus*, are essential to induce diurnal *Reg3γ* expression in a MyD88-dependent fashion [120]. REG3γ diurnal dynamics also appear to be responsible for rhythmic patterns of bacterial adherence to the proximal colon mucosal surface, which corresponds to rhythmic expression of metabolic genes within the intestinal epithelium [121]. GF mice colonized with mouse-derived gram-positive segmented filamentous bacteria (SFB) exhibited diurnal rhythmicity in bacterial abundance and localization, and also had restored oscillatory gene transcriptional patterns in the host colon resembling those of SPF mice, e.g., genes related to DNA replication, cell cycle, and nucleotide turnover [121]. This suggests that the effect was specific to gram-positive bacteria. The oscillatory transcriptional effects of SFB colonization of WT GF mice were also blunted in *Reg3γ* deficient mice. Interestingly, the colonic transcriptional profiles of ABX-treated SPF mice resembled those of *Reg3γ* deficient mice. These studies demonstrate the intricacy of circadian crosstalk between microbes and the local, intestinal host immune system and metabolic outcomes.

### 6.5. Melatonin

Melatonin is a hormone produced throughout the intestine by ECs, and binding of melatonin to EC membrane receptors triggers signal transduction pathways [122]. Few experimental studies have assessed circadian host–microbe interactions involving melatonin, but evidence suggests melatonin may induce diurnal oscillations of gut microbes in ways that are relevant to host metabolism. For instance, administration of melatonin to mice produced similar oscillations of core clock genes in both HFD and control-fed mice, which correlated with specific diurnally oscillating microbes and host lipid indices [123]. Another study showed that a specific bacterial species (*Enterobacter aerogenes*) isolated from human stool responded to melatonin administration in vitro by changing its swarming behavior in a circadian manner [124]. This was unique to melatonin and did not occur after treatment with structurally similar compounds or in other bacterial strains tested. Further studies are needed to identify how melatonin contributes to circadian dynamics in gut microbiota in vivo. However, the majority of preclinical studies describing gut microbial oscillations use mice on a C57Bl/6 background, which are inherently melatonin deficient due to genetic biosynthesis pathway defects [125]. Studies using a congenic C57Bl/6 line [126] or outbred rodent strains may provide further insights. Regardless, these studies suggest specific host-derived hormones can induce diurnal rhythms in activity of specific gut microbes [124], which may in turn contribute to rhythmic changes in host nutrient absorption.

## 7. Gaps and Challenges in Defining the Role of Host–Microbe Rhythmic Orchestrations

### 7.1. Defining Additional Orchestrators and Conductors of Microbe-Mediated Circadian Rhythms

The coordination of CRs between gut microbes and host immune-mediated control of metabolism is crucial to maintain homeostasis and prevent or alleviate metabolic dysregulation. While rapid advances of knowledge in these areas have been achieved, many of the observations are associative; key questions remain in order to advance the field to identify causation and translate these findings to the clinic. One key question regarding gut microbe diurnal oscillations is “What Zeitgebers drive these phenomena?”. While timing of enteral feeding and nutrient delivery to the intestine is a primary Zeitgeber, one study noted parenteral feeding (constant intravenous infusion of nutrients) in mice selected for unique microbial community membership that still exhibited diurnal oscillatory behavior [38]. This suggests direct intake of nutrients through enteral feeding is not the only cue promoting gut microbial oscillations, indicating intrinsic host factors may also contribute to microbial community dynamics [38]. For instance, features of the intestinal mucosal environment, including host innate immune components made by IECs, such as AMPs, exhibit diurnal rhythms independent of the core clock and provide signals that drive oscillations in specific gut microbial community members [120,121,127]. Identification of additional innate and adaptive mucosal immune components that aid in maintaining local, intestinal rhythmicity in gut microbes and serve as a conduit of communication is essential to identify host targets that can be used to promote circadian gut eubiosis. A great deal of preclinical studies have used stool and luminal samplings from other intestinal regions (e.g., cecal contents) to describe microbial rhythms, however, mucosa-associated populations may be more relevant to host parameters, such as receptors, transporters, and immune cells, associated with metabolic and immune-mediated outcomes.

### 7.2. Orchestrations of Host–Microbe Circadian Rhythms in Humans: Association vs. Causation?

While preclinical models have made studies of host–microbe circadian dynamics possible, the translation of these findings to humans is limited and remains associative in many instances. The first insight into microbes exhibiting diurnal patterns in humans that corresponded to circadian misalignment was shown in two individuals, where stool samples were collected at different times of day over several days before and after acute jet lag [40]. Similar to observations in mice, ~10% of the microbial community, determined via 16S rRNA gene sequencing, exhibited oscillations, including taxa belonging to *Parabacteroides*, *Lachnospiraceae*, and *Bulleida*, while 20% of the functional characteristics determined via shotgun metagenomics were rhythmic under baseline conditions. Eight to ten-hour phase advance due to travel promoted an expansion of Firmicutes, which normalized to baseline after 2 weeks. Conventionalization with 1 day pre- and 1-day post jet lag microbes into GF mice revealed increased weight gain and loss of glucose tolerance from jet lagged-induced microbial transplant. Changes associated with pre- and post-jet-lagged microbiomes in immune parameters, such as cytokines associated with dysregulated metabolism were not examined either in the human microbiome donors or in the GF recipient mice.

Given the inherent difficulty of collecting repeat stool samples from individuals within a 24 to 48-h period, several studies have relied on 16S rRNA gene sequencing from saliva. While one study did not identify distinct 16S rRNA gene diurnal rhythms in saliva collected every 4 h over one day from 5 healthy subjects [128], Takayasu and colleagues were able to identify significant oscillations in ~79% of the microbial community, including the genera *Peptostreptococcus*, *Streptococcus, Solobacterium, Haemophilus*, and *Gemella* belonging to the Firmicutes phylum as well as the genus *Prevotella* of the phylum Bacteroidetes in samples collected from 6 healthy subjects at an identical time interval over 3 days [129]. Furthermore, oscillations could be grouped based on bacterial phenotypes (aerobic, anaerobic, facultative, Gram positive vs. Gram negative) and none of the observed rhythms could be recapitulated following in vitro culture. Functional pathway enrichment determined via metagenomics analysis also revealed diurnal rhythms in 55 KEGG modules associated with bacterial carbohydrate and lipid metabolism and environmental information processing, amongst others. These observations, while insightful, remain associative and host factors contributing to these changes are unclear. Through addition of an intervention, Collado and colleagues explored whether taxonomic community membership oscillations in saliva were dependent upon time of eating, where 10 healthy women ate an early or late lunch followed by a washout period in a crossover design [130]. Here, diet was standardized across subjects and sleep diaries were recorded along with collection of 4 saliva samplings over a 24-h period coupled with a single stool collection following each arm of the study. This study revealed food timing did not impact overall microbial community membership, however, alpha diversity indices were different dependent on sampling time. Early eating promoted diurnal rhythms in the phylum TM7 as well as the genus *Bacteroides, Ruminoccocaceae* and *TM7*-3, while late eating exhibited rhythms in several members of the phylum Fusobacteria. Together, the random crossover study design supports the notion that meal timing can directly influence diurnal patterns of a human-derived microbial community. Additional studies using interventions, even those using a small number of well-phenotyped subjects, can help to gain insights into the diurnal nature of gut microbes in health and disease.

Despite this knowledge, the application of diurnal variation in gut microbes to a broader population remains unclear. Given the vast amount of gut microbiota variability between humans, it is difficult to determine how oscillations of one individual’s microbes relates to another individual’s, particularly if they have distinct chronotypes. Recent studies have used timestamped stool collections to “reconstruct” microbial rhythmicity. In one study, 3 timestamped baseline samples from 28 healthy men and women were collected over 3 days prior to onset of a diet intervention. A retrospective look at SCFA levels showed an association with time of sample collection and relative abundance of ~35% of taxa, where meal timing and overnight fasting duration also correlated [131]. When all factors were included in the linear mixed model statistical analysis, relative abundances of *Bifidobacterium*, *Butyricimonas*, *Sutterella*, and *Bilophila* increased, while *Collinsella*, *Streptococcus*, and *Eubacterium* decreased throughout the day. One limitation in this study that persists across many human studies is the collection of stool samples occurring only during normal waking hours with no overnight sampling. This prevents application of standard 24-h statistical approaches, such as JTK_cycle. Coupling similar studies with a preclinical model, such as conventionalization of gnotobiotic animals with participants’ stool and feeding the animals with diets that mimic the participants’ dietary consumption could allow for a more robust interrogation of both microbial oscillations as well as rhythms in host parameters, such as those involved in metabolic and immune signaling.

Further capacity to reconstruct microbial rhythmicity in large datasets was recently shown, where time of stool collection strongly correlated with 16S rRNA gene microbial community membership in the KORA cohort, consisting of ~2000 individuals from the same region of Germany that contained both healthy subjects and those with metabolic diseases, such as T2D [132]. By pooling single stool samples over a 24-h period, this work revealed oscillations of the dominant phyla Firmicutes and Bacteroidetes as well as of specific genera in healthy subjects; most intriguingly, the amplitude of these oscillations was either dampened or lost in pre-diabetes and T2D. Here, rhythmicity in 13 taxonomic features was lost, including *Bifidobacterium longum, Escherichia coli, Eubacterium rectale, Fecalibacterium prausnitzii, Intestinibacter bartletti, Clostridium celatum,* and *Romboutsia ilealis*. Shotgun metagenomics in a subset of samples revealed significant associations between 26 functional microbial pathways corresponding to the T2D arrhythmic microbial signature. Whether oscillations in these microbial features played a causative role in T2D was not clear from these data; however, application of machine learning techniques using these 13 arrhythmic taxa showed their capacity to identify T2D in regionally similar large cohorts, suggesting the possibility of using dysregulation of microbial rhythms in predicting certain disease states.

### 7.3. Identifying the Molecular Underpinnings of Host–Microbe Circadian Rhythms

Many preclinical and clinical studies rely on gene amplicon sequencing-based techniques to examine oscillations in the microbiome. These data are plagued by analyses of relative abundance, which can skew or inadequately report rhythms, as previously shown by Liang and colleagues [61]. In this study, inferred absolute abundance of 16S rRNA copy number suggested that oscillations in the phylum Bacteroides were comparable to those determined via relative abundance. In contrast, inferred absolute abundance analysis revealed no rhythms in Firmicutes, while gain of rhythms in Proteobacteria were observed which was incongruent with relative abundance data analyses. A modality to more precisely correct for absolute abundance is needed to improve the application of statistical tools to biological rhythms. Furthermore, the sequenced variable region of the microbial gene, e.g., V3-V4 region of the 16S rRNA gene, must be reported, and for large cohort datasets where validation is essential, similar variable regions should be compared. Finally, rhythms in additional microbial community members, such as fungi, should be considered. Existing circadian shotgun metagenomic datasets could be leveraged to examine sequences associated with additional, but less abundant, kingdoms of the intestinal microbial community. Network analyses and modeling tools could then be applied to identify key functional characteristics of the microbiome that impact circadian dynamics of host immunometabolism. By integrating sequencing and bioinformatics-based approaches, classical microbiology techniques, and other in vitro and in vivo preclinical models, the precise roles of specific microbial functions in driving oscillations and downstream metabolic and immune parameters can be determined. Application of multiple tools and techniques to examine microbial rhythms will permit in-roads into identifying key features and mechanisms involved in maintaining host immune-mediated metabolic homeostasis.

## 8. Conclusions: The Score Continues in the Orchestration of Host–Microbe Circadian Rhythms

In summary, identification of the transkingdom interconnections between host and microbe circadian networks has provided a novel angle from which to approach the microbiome’s mechanistic role in maintaining host health. Dysregulation of circadian dynamics in any member of this mutual relationship elicits a dramatic impact on host metabolism. While the mechanistic underpinnings of these interactions are an active area of investigation, it is apparent that the host immune system plays a crucial role in relaying information between host and microbial systems. Understanding additional components that contribute to circadian transkingdom alignment may lead to identification of novel, molecular mediators that can be targeted for preventing or treating metabolic diseases.

## Figures and Tables

**Table 1:** Connections between environmental or genetic disruption of circadian rhythms (CRs), gut microbiota, metabolism, and immunity.

CRs Disruption	Gut Microbiota	Metabolism	Immune System
Jet lag	- Increased gut permeability [49,50] - Changes in overall fecal community membership and loss of microbial oscillations [40,49,51] - Downregulation of SCFA production [52]	- Increased body weight and adipose tissue content [40,49,51] - Altered food and water intake patterns [40,51,53]	- Increased levels of inflammatory macrophages and decreased T regulatory cells in adipose tissue [51] - Elevated intestinal Th17 cell number [54] - Endotoxemia [55]
TRF (active phase)	- Restored gut microbiota community disruption and loss of oscillations induced by HFD [39]	- Restored transcript oscillations in liver and protects against weight gain on HFD [56]	- Did not impact adipose tissue inflammation and macrophage infiltration [57] - Increased expression of serum cytokines [58]
TRF (rest phase)	N/A	- Disrupted normal feeding and hepatic transcription patterns [59]	- Disrupted diurnal difference in helminth expulsion rate [60] - Reduced expression of serum cytokines and bacterial killing in response to LPS challenge [58]
*Bmal1*	- Altered taxonomic composition of fecal microbial community [61]	- Arrhythmic and reduced activity [10] - Impaired glucose and lipid homeostasis [13,14] - Insulin insensitivity [62]	- More susceptible to infection [63] - Loss of rhythmic lymphocyte trafficking [64] - *KO in ILCs*: reduced ILC numbers in intestine [65] - *KO in myeloid cells* [18], *neutrophils* [66], *lymphocytes* [64]: abolished rhythmic trafficking, reduced infection clearance rate
*Clock*	- Increased gut permeability [50] - Altered fecal microbial community and reduced taxonomic diversity [67]	- Increased body weight and food intake [68]	- Ameliorated induction of dermatitis via IL-23 receptor [69] - Reduced expression of proinflammatory genes; reduced intestinal colonization of *Salmonella* [70]
*Cry1/2*	N/A	- Increased weight gain and lipid uptake, and hyperinsulinemia [71]	- Autoimmune response (increased IgG concentration, leukocyte infiltration) [72] - NF-κB activation [73] - Increased activated T cells and TNF-α production; exacerbated joint swelling in arthritis model [74]
*Per1/2/3*	- Altered fecal microbial community and loss of oscillating taxa [40]	- Leptin resistance [75] - Altered lipid metabolism [76] - Loss of diurnal feeding rhythm [77] - Increased adipose tissue; Decreased muscle tissue [78]	- Loss of daily rhythms of IFN-γ in spleen and serum [79], and cytolytic natural killer cell factors [80] - Protection from LPS-induced mortality [81]
*Rev-erbα/β*	N/A	- Altered lipid homeostatic gene networks and metabolism; altered wheel-running patterns [82] - Reduced body fat and increased lean mass via *Nfil3* repression [83]	- Loss of diurnal endotoxin response [84] - Disruption of Th17 cell lineage specification by *Nfil3* suppression [54] - Loss of signaling relay between ILCs and *Nfil3* [83]
